# Design and Development of Layered Security: Future Enhancements and Directions in Transmission

**DOI:** 10.3390/s16010037

**Published:** 2016-01-06

**Authors:** Aamir Shahzad, Malrey Lee, Suntae Kim, Kangmin Kim, Jae-Young Choi, Younghwa Cho, Keun-Kwang Lee

**Affiliations:** 1 Center for Advanced Image and Information Technology, School of Electronics & Information Engineering, Chon Buk National University, 664-14, 1Ga, Deokjin-Dong, Jeonju, Chonbuk 561-756, Korea; aamirshahzad@gmail.com; 2 Department of Software Engineering, Chon Buk National University, 664-14, 1Ga, Deokjin-Dong, Jeonju, Chonbuk 561-756, Korea; stkim@jbnu.ac.kr; 3 Division of Biotechnology, College of Environmental & Bioresource Sciences, Chonbuk National University, Iksan 570-752, Korea; 4 College of Information and Communication Engineering, Sungkyunkwan University, Suwon 440-746, Korea; choyh2285@skku.edu; 5 Department of Beauty Arts Care, Koguryeo College, Naju 520-930, Korea; kklee7410@hanmail.net

**Keywords:** supervisory control and data acquisition, distributed network protocol, dynamic cryptography buffer

## Abstract

Today, security is a prominent issue when any type of communication is being undertaken. Like traditional networks, supervisory control and data acquisition (SCADA) systems suffer from a number of vulnerabilities. Numerous end-to-end security mechanisms have been proposed for the resolution of SCADA-system security issues, but due to insecure real-time protocol use and the reliance upon open protocols during Internet-based communication, these SCADA systems can still be compromised by security challenges. This study reviews the security challenges and issues that are commonly raised during SCADA/protocol transmissions and proposes a secure distributed-network protocol version 3 (DNP3) design, and the implementation of the security solution using a cryptography mechanism. Due to the insecurities found within SCADA protocols, the new development consists of a DNP3 protocol that has been designed as a part of the SCADA system, and the cryptographically derived security is deployed within the application layer as a part of the DNP3 stack.

## 1. Introduction

Supervisory control and data acquisition (SCADA) systems have been accorded a prestigious status and play important roles within the real-time industrial processing and automation fields [1,2]. Along with the massive changes in the field of information technology (IT) and the increasing use of IT by humans throughout their daily lives, SCADA systems have also changed from simple stand-alone systems, or “relay logic”, to network-based systems like a traditional computer network [2,3].

Typically, a SCADA system is governed by a central controller or main controller that is a part of its hierarchical network structure that is designed and configured to comprise several remote stations or remote terminal units (RTUs); these remote devices collect information from physical devices and transfer the information back to the main controller for scrutiny and control purposes [3,4].

SCADA-system protocols were originally designed and used as proprietary protocols; each protocol was developed by a specific manufacturer for a specific industry as a part of a proprietary SCADA system to fulfill the basic needs of that specific industry [3,4]. With the increasing demands on SCADA systems and the underlying interoperability problems, the need for non-proprietary protocols was identified [4]. Today, many open-standard protocols such as distributed network protocol version 3 (DNP3), Modbus, and Fieldbus have been developed by SCADA organizations, and have resolved the dilemma of interoperability by providing connectivity for the devices and equipment of different manufacturers and vendors [5]. Given the evolution of the open-standard protocols that are used within SCADA communication, users can now purchase and use equipment such as master terminal units (MTUs), RTUs, and other physical devices from a variety of manufacturers. This larger interconnectivity between a number of open-standard protocols and the proprietary protocols, however, has resulted in the heightened vulnerability of SCADA systems to several types of security attacks [6,7].

DNP3 is a conventional protocol that has been used in SCADA industrial processing including electricity generation and distribution and water distribution. DNP3 is an open-standard protocol that is built on a three-layered, enhanced-performance-architecture (EPA) model. The DNP3 protocol contains the following three layers: the application layer, data-link layer, and physical layer. One additional layer, called the “pseudo-transport layer”, is added to the DNP3 stack to permit the transmission of enormous quantities of data [8]. The DNP3 protocol facilitates reliable communication between the SCADA nodes [8,9]. The proposed study emphasizes not only the application layer in the DNP3 protocol-development process, but also the vulnerabilities that have arisen at an application level, regarding the message transmission to/from the SCADA/DNP3 protocol, and regarding TCP (transport control protocol)/IP (internet protocol) communication over the Internet.

## 2. Problem Statement

The details of the vulnerabilities and security issues that affect SCADA communications were reviewed and analyzed [10,11,12]; based on this review, an appropriate, flexible, and protective security solution for SCADA communication could not be identified. Each of the possible security solution has limitations due to its dependency on other security mechanisms and protocols [13,14,15].

A SCADA system’s potential security challenges exist because of the larger interconnectivity issues between proprietary and non-proprietary protocols and the use of modern IT [15,16,17,18,19,20,21,22]. A generic security solution is desirable for the resolution of security issues such as confidentiality, authentication, integrity, and non-repudiation [23,24,25,26,27]. Established global organizations including the U.S. National Security Agency, U.S. Homeland Security Department, Schweitzer Engineering Laboratories, Inc., the American Gas Association, and the Gas Technology Institute, have been vulnerable to security threats due to the Internet’s open connectivity [28,29]. These organizations and other researchers have developed several security solutions and protocols to guard against cyber-security issues, and the cryptography-based security mechanisms have been selected as the “best” security approaches for secure SCADA communications [26,30,31,32,33,34,35,36,37,38,39,40,41,42]. As a consequence, the DNP3 users group defined and described the cryptography approaches, such as the asymmetric and symmetric methods, to enhance the security of the DNP3 protocol; therefore, the details of several cryptography algorithms are available along with the information regarding their security parameters [43,44,45,46,47,48,49,50,51,52,53,54,55,56,57]. The main parameters such as integrity and authentication reportedly ensure and secure communication at an application level by employing a challenge-response mechanism and are significant in their protection against spoofing, modification, and replay attacks [8,58]. Several limitations, however, are acknowledged regarding the design of the DNP3 secure-authentication mechanism (most of the work remains under development) including a capability that only ensures end-to-end security rather than protocol-embedded security [55,56,57].

Supreme security protocols and/or solutions, such as secure sockets-layer (SSL)/transport-layer security (TLS), Internet protocol security (IPSec), secure shell (SSH), and key agreements and management, among others [43,44,45,46,47,48], have been deployed within traditional network systems and/or SCADA systems; however, these solutions have several limitations due to the protocol dependency, an end-to-end-security focus, and a reliance upon the other cryptography protocols for security [5,14,49,50]. An actual security enhancement was made to conquer the security issues that usually reside in the transmissions of the SCADA system; in this enhancement, the DNP3 protocol is considered, and encryption and authentication mechanisms that modify the original frame of the DNP3 protocol are employed. The cyclic-redundancy-check (CRC) bytes are replaced with new security fields that are designated to compute the security via the cryptography mechanism. The scope of the development, however, is limited to a data-link layer, and the security development is also limited to only the provision of a stronger authentication and non-repudiation security parameters for the DNP3 protocol [59].

In conclusion, an inclusive security solution is required rather than end-to-end security to resolve these limitations and enhance the security of SCADA/DNP3-protocol communications [5,14,25,30,32,45,51]. A security solution or model has therefore been proposed to supplant end-to-end deployment and to enhance the security of the DNP3 protocol as part of a critical, infrastructural communication (or SCADA)-system-using, protocol-embedded security.

## 3. Scope of Study

**Figure 1:**
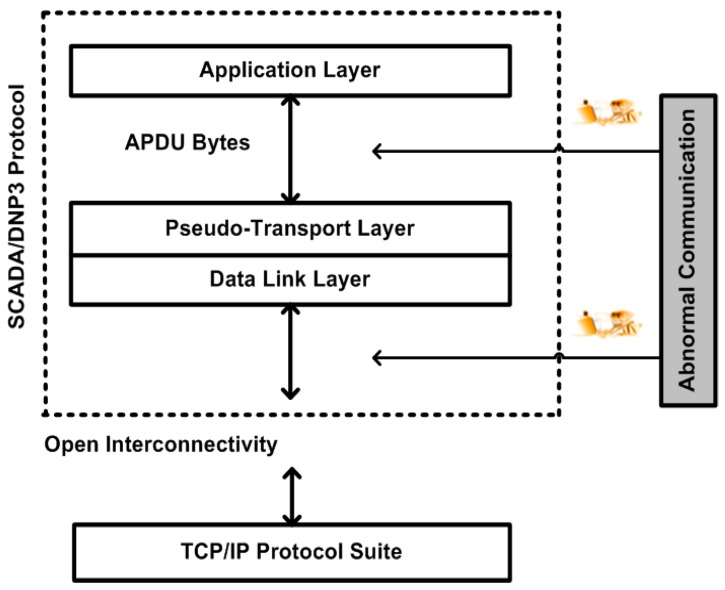
Research scope and study gap.

In this study, a DNP3 protocol stack was designed and an inclusive security solution that uses cryptography algorithms such as AES, RSA, and SHA-2 is solely deployed at the DNP3-protocol-application layer; however, end users were allowed to deploy and test the other cryptography algorithms. The application layer is considered the most sensitive layer in the DNP3 protocol because the protocol message is generated at the application-layer level when a message is designated as sent or received, and the message is then passed to a lower level for further execution [8,10,58]. This study therefore shows the deployment of security at the application level, or the deployment of application protocol data unit (APDU) security as a part of the application layer, before the APDU bytes are transmitted to the lower layers of the DNP3 protocol as a part of the SCADA system (security) and/or open protocols. Further details are shown in Figure 1, as follows.

## 4. Research Objectives

The main objectives of this study are as follows:

1. The bytes of the application layer or the APDU bytes are fixed at the application level; the dynamic cryptography buffer (DCB) is used for the remaining bytes after the fixing process is complete. The APDU bytes are therefore readily aligned with the pseudo-transport-layer bytes or the TPDU bytes during the transmission of the message or the bytes.
2. The assemblage of the protocol bytes from the lower layer to the upper layer, whereby security is implemented and tested in terms of integrity, authentication, confidentiality, and non-repudiation at the application layer of the DNP3 protocol.
3. The proposed security development is validated using formal proofs and the performance results are evaluated with security tools. The security results are also measured during the SCADA/DNP3-protocol end-to-end communication for the purposes of a performance comparison.

## 5. Research Contribution

The contribution of this study is three-fold, as follows: a new security mechanism was designed and implemented within the original SCADA/DNP3-protocol stack; the bytes are constructed at each layer, flowed within the stack, and transmitted to/from the TCP/IP protocols at both sides or on the send/receive side.

The application-layer bytes are aligned with the pseudo-transport-layer bytes within the DNP3 stack; the remaining 56 bytes are used for the DCB. The DCB was designed and deployed to keep track of the bytes during message construction and security implementation. The new security mechanism was implemented and tested using cryptography to enhance the security of the SCADA/DNP3-protocol communications, and it also provides a platform to test and implement other cryptography approaches (or algorithms).

The security mechanism was designed and implemented according to the requirements of SCADA/DNP3 unicasting communication. Formal methods and tools have been used to validate the proposed security development and to evaluate its performance. The overall performance results were measured and evaluated after the verification of formal proofs.

## 6. Development of the Application Layer

The application layer is the highest or top layer of the DNP3-protocol stack that interacts directly with user-application programs or takes data/messages from the user-application interface/program. The application layer interacts with the user-application program and accepts user-requested data/messages depending on the requirements of the user. Data may be in any form such as digital, analog, events (or the actions performed for an event), alarms, reports, and data files. Data size is not limited in the DNP3 protocol; the DNP3-application layer takes random data from a user-application program and manipulates this data. The application layer converts the data from the user-application program into manageably sized blocks. These blocks are also referred to as application service data units (ASDUs), and each ASDU size is limited to 2046/2044 bytes in the case of a request/response [2,9].

The application layer creates APDUs by combining or adding an application header that is called “application protocol control information” (APCI). The APCI fields consist of two or four bytes depending on whether the data requests a reply back. In the proposed implementation, the APDU size is limited to 1992 bytes; if the requested/response data is larger than 1992 bytes, then more than one APDU will be created, depending on the size of the user data. In the original DNP3 documentation, the numbers of ASDU blocks are not limited but each APDU’s size is limited to 2048 bytes. The APDU-buffer size is sufficient to process the entirety of the information in the data requested/received and, after processing, the buffer is discarded for the next APDU bytes [9].

The total number of user blocks in the ASDU is eight. Each block has 249 bytes of information that have been accepted from the last block because the last block has 247 bytes in the case of a request and 245 bytes in the case of a response. These blocks readily fit into the transport-protocol data units (TPDUs) of the pseudo-transport layer. A DCB is created based on the remaining 56 bytes from each ASDU and is used for storage information that is related to cryptography deployment and other service information for the development of the testbed.

**Figure 2:**
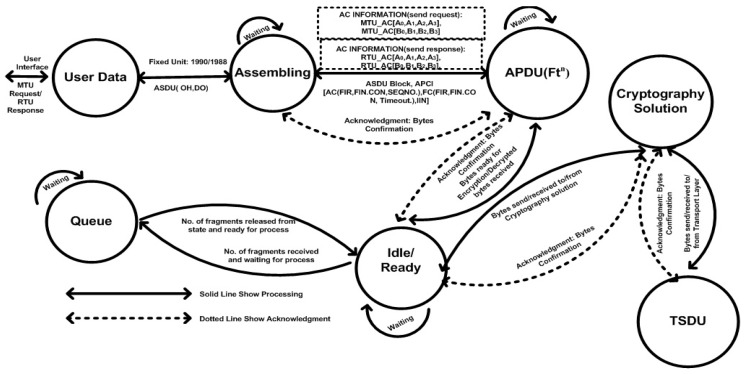
Application-layer state-transition process with cryptography solution.

The fixing process for the ASDU bytes is two-fold, as follows: the APDU bytes are aligned with the TPDU bytes and the remaining 56 bytes could be used for cryptography deployment within the application layer of the DNP3 protocol. When the APDU is constructed in the application layer, the control is passed to the cryptography solution for security deployment. In Figure 2, the master terminal unit (MTU) generates the request message and sends it to the remote terminal unit (RTU) that then generates the application-level response to the MTU after it receives the request. In the case of a command execution between the MTU and RTU, the header is only transmitted without any ASDU bytes. The following two types of message headers are in the application layer of the DNP3 protocol: request and response headers. The response header has an additional two-byte field called the internal indication (IIN) that indicates the difference between the request and the response headers in the APCI.

In the application layer, the MTU is responsible for sending request messages to the RTU and the RTU is responsible for sending the response according to the MTU request; however, the DNP3-application layer has a special class of response that is used by the RTU for the purpose of sending information to its predefined master station. This special class of response is also called an “unsolicited response” and is typically based on the critical events or process changes that are detected within a communication.

In Figure 2, the highlighted part shows the confirmation set (bit) that is transmitted between the master terminal unit (MTU) and remote terminal unit (RTU) and/or vice versa. The application control (AC) is accounted as a main field of application layer header which used to control the communication between the nodes. AC contained four subfields, such as first fragment (FIR), final fragment (FIN), confirmation (CON), and sequence number and in the below communications 1 and 2 these subfields are represented in the form of [A0, A1, A2, A3] and [B0, B1, B2, B3], with distinguished set bit (*i.e.*, CON or A2/B2 = 0 or 1). More detail is provided in the subsequent part of this paper, as follows:


**MTU/RTU Communication 1 {**
If Request: MTU_AC [A0, A1, A2 = 0, A3] && Response: RTU_AC [A0, A1, A2 = 1, A3]
Then Confirmation: MTU_AC [A0, A1, A2 = 0, A3]
**}** // Master station has initialized the request without confirmation and upon receiving the RTU response with a confirmation set (bit), the MTU also sends the confirmation.



**MTU/RTU Communication 2 {**
If Request: MTU_AC [B0, B1, B2 = 1, B3] then Confirmation: RTU_AC [B0, B1, B2 = 0, B3]
&& Response: RTU_AC [B0, B1, B2 = 1, B3]
Then Confirmation: MTU_AC [B0, B1, B2 = 0, B3]
**} //** Master station initialized the request with the confirmation set (bit) and the RTU also sends the confirmation message. Afterward, the RTU will send a response with a confirmation set (bit) upon receipt and the MTU also sends the confirmation to the RTU.


When the MTU sends a request message to the RTU with a confirmation bit, upon the receipt of the message, the RTU also sends an acknowledgment (message) to the MTU. The RTU assembles the necessary information related to the requested message; once the message is ready, then it is sent to the MTU. The MTU also sends a confirmation (message) if the confirmation bit is set in the response (message).

In a SCADA system, communication between field devices is either in the form of commands or data. Usually, the RTU will generate the data response according to the MTU commands. The message or data always contains specific data objects and function codes that are added at the start of a communication-message request or response. The SCADA/DNP3 protocol provides reliable communication between different vendor’s devices and makes data meaningful across several platforms. The following sections are the core application-layer functions that have been used during message construction, and the types of information such as the function code and data objects that are involved in communication.

### 6.1. Application Protocol Control Information

**Figure 3:**
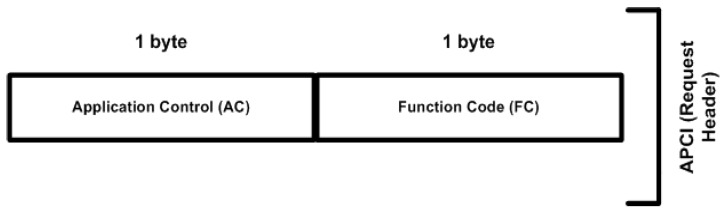
Request header structure.

**Figure 4:**
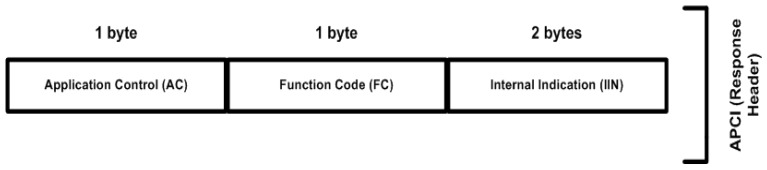
Response-header structure.

The application header is also called the application protocol control information (APCI). APCI has two-byte fields for a request header and a four-byte length field for a response header, whereby the request and response headers differ by two bytes in the application layer. The response header has an additional two-byte field, designated as the IIN, which distinguishes between the request and response headers in the APCI. Figure 3 shows the APCI or the case of APCI bytes being sent, while Figure 4 shows the APCI or the case of an APCI-byte response.

The first byte of the APCI request or response is designated as the application control (AC). The communication between the MTU and RTU is controlled by the AC field. The AC field carries one byte of information, having three bits or flags, such as FIR, FIN, and CON, and a five-bit sequence number that is used to move fragments (or APDUs) in a sequential order.

In MTU/RTU communication, if the MTU sets the confirmation bit as CON = 1 within the requested message, then the RTU will also be responsible for sending a confirmation within the response message with the same sequence as the MTU-requested sequence number. In a case where multiple messages are received by the MTU/RTU with the same sequence number, then the second number will be ignored and the first one will be accepted; if nothing is received by the MTU/RTU, then the request will be transmitted again. The sequence number is incremented with the APDU increments, and the response-APDU sequence number is the same as the requested-APDU sequence number.

The first APCI field is used to manage the communication flow and the second field is designated a function code (FC); the FC carrys one byte of information that is used to define the actual meaning of the message/data to be sent/received between the MTU and RTU. Data is the most important part of SCADA/DNP3 communication. As previously mentioned, the DNP3 protocol is designed to manipulate different types of data, such as digital, analog, event or an action performed for an event, alarms, reports, and data files. The DNP3 protocol not only provides reliable communication between field devices that are connected in a SCADA network, but it also makes the data meaningful across different networks to understand the meaning and purpose of the message.

In the second APCI field, the function codes are used to define or identify specific functions for the data that are being operated or the function that is used to perform with the data. Some function codes are limited to the specific data on which they operate; therefore, data-object references are also included within the ASDU.

The IIN field that is used in the response header (or APCI) contains two bytes of information followed by a function code, and it is used for sending a response to a desired station. Each bit inside the IIN field has a specific meaning for the purposes of response-message generation, usually from the RTU to the MTU. The RTU-response message defines the flags that correspond to the IIN field that are copied in the message each time the message is sent from the substation to the master station. Using the IIN field in the response header, the substation will then report to the master station.

In a SCADA system, the sent/received message carries commands and sometimes data as a response from the RTU, whereby the data is composed of function codes and data objects. The FC defines the meaning and uses of the bytes (or message) in the DNP3 protocol and the data objects define the structure and the data interpretation process.

### 6.2. Application Service Data Unit

As discussed, there are two types of data/message communications within a SCADA system such as a request from the MTU, a response from the RTU, and/or an unsolicited response. Each time, one or more APDUs are created, depending on the size of the user data that is being requested/responded to. If more than 1992 bytes with a header are sent from the MTU, then multiple APDUs are generated and transmitted in a sequential order. Each APDU is represented as a fragment with fields such as APCI and ASDU. The ASDU is made up of data objects and each data object is followed by an object header. In the ASDU field, the proposed research logically fixed the number of data blocks at 8 and each block is 249 bytes, with the exception of the last block; furthermore, the proposed research logically fixed the ASDU size up to 1990 bytes in the case of a request and 1988 bytes in the case of a response.

Here, each data block (or block) of an ASDU contains an object header and data objects. Through logical fixing of the ASDU, the data blocks easily fit into (or align with) the transport protocol data units (TPDUs) in the pseudo-transport layer of the DNP3 protocol; for example, the 1992 bytes of APDU are transmitted and are treated as user bytes in the pseudo-transport layer. The bytes of the transport service data unit (TSDU) are assembled and then converted into 8 data blocks, and 1 byte of header information is added to each data block resulting in the formation of 8 TPDUs. Because the lower layer (or data-link layer) only carries up to 250 bytes from the pseudo-transport layer, the logical fixing process of the ASDU is two-fold, as follows: 1) The APDU bytes (or 1992 APDU bytes) are aligned with the TPDU bytes in the pseudo-transport layer. 2) The remaining 56 bytes (from the total of 2048 APDU) are used for the DCB. In the original DNP3 documentation, the user-data blocks are not fixed.

**Figure 5:**
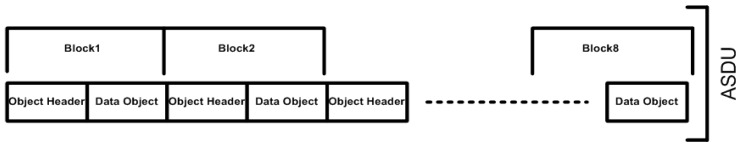
New logical fixing of ASDU.

In the ASDU, each data block is composed of the following two subfields: object header and data object. Each ASDU field is composed of one or more data blocks, but the blocks are logically limited to 8 (blocks) and the maximum ASDU size is up to 1990 bytes/1988 bytes in the case of a request/response. Figure 5 shows the request/response ASDU structure (or new, fixed ASDU blocks) with subfields such as “object header” and “data object”.

The object header specifies the data-object type and the instance of data that is being referenced by the message. The object header is made up of the object, qualifier, and range fields between 3 bytes and 11 bytes of length.

In the ASDU, “object header”, “qualifier”, and “range” fields are used to identify the specific data points of each object group, and the variations that are being referred to as data are used on both the sender and receiver sides. In a request message, only the data identification will be required and data objects are included in response messages from the substation. The qualifier field is composed of the qualifier code or “Q-code”, the index size or “I-size”, and provides the qualifier-code value. The qualifier field with the range field works to fully identify the data objects that follow each object header in the ASDU. In the object header, the object field is subdivided into two fields such as the object group that defines the general type of data and the object variation that defines a particular variation of data and up to 2 bytes of length.

### 6.3. System Model: Application Layer Development

This study used the open library of the DNP3 protocol and the explicit codes were deployed, thereby finalizing the proposed system’s design and development. To validate the proposed application-layer (message) design and development, Postulate 1 to Postulate 4 were employed, and these are further used for the verification of the algorithm and during the security-development phase. The basic notations that are used in this section are summarized in Table 1.

**Table 1:** Terminologies for system design and development.

Notations	Descriptions
X	*Random bytes from user-application layer or random input bytes from user-application layer.*
$f_{µ}$	*Function that defines bytes X*
n	n=1,2,3,…,k,wherekisalimitedinteger
$f_{APCI}(X)$	*Function that computes the application-layer header bytes or APCI bytes.*
$f_{ASDU}(X)$	*Function that computes the application-layer data bytes or ASDU bytes.*
$\left(X_{h},X_{d}\right)$	*Computed header (h) bytes* $X_{h}$* and data (d) bytes* $X_{d}$*.*
$X_{dy}$	*Computed dynamic (dy) bytes* $X_{dy}$ *via function* $f_{dy}$*.*
$f_{APDU}(X)$	*Function that computes the application layer fragment or APDU bytes. Such that* $f_{APDU}(X)=f_{APCI}(X)+f_{ASDU}(X)$*.*
α∈(S;R)	*Distinct identifier(s) for sender(S) or responding (R) message (M)/bytes (b)*
$\left(f_{AC},f_{FC},f_{IIN}\right)$	$f_{APCI}(X)$ *or APCI bytes that are computed by functions:* *$f_{AC},f_{FC},f_{IIN}$, in the case of sending(S)/responding(R).*
$\left(x_{1},x_{2},x_{3}x_{4},x_{\left(im,ex\right)},x_{iin}\right)$	*Parameters of function* $f_{APCI}(X),s$*. Such that,* $f_{AC},f_{FC},f_{IIN}\in f_{APCI}$.
$\left(f_{OH},f_{OD}\right)$	$f_{ASDU}(X)$ *or ASDU bytes that are computed by the following functions:* $\left(f_{OH},f_{OD}\right)$, *in the case of sending(S)/responding(R).*
$\left(x_{5},x_{6},x_{7},x_{8}\right)$	*Parameters of function* $f_{ASDU}(X)$*, such that* $f_{OH},f_{OD}\in f_{ASDU}.$
$\left(x_{5.1},x_{5.2}),(x_{6.1},x_{6.2}\right)$	$\left(x_{5.1},x_{5.2}\right)$ *are implied for object function(of) and* $\left(x_{6.1},x_{6.2}\right)$ *are implied for object qualifier (* oq *)*
$f_{L}(X_{d})$	*Function that logically fixed the ASDU bytes into data blocks (DB).*

*Postulate 1*.  The number of bytes *X* is defined and received from the user-application layer through the use of function $f_{µ}$. The function $f_{APDU}\in f_{µ}$ exists and is performed at both sides of the transmission with the usage of the distinct identifiers α∈(S;R), and simultaneously interacts with and is updated in the dynamic storage as a part of the protocol stack.

The random bytes X are retrieved from the user-application layer by performing the function $f_{µ}$ that is designated to check the bytes X that are limited by the proposed development. In the case of the sending (S)/responding (R) APDU bytes, the functions $f_{ASDU}$ and $f_{APCI}$ are employed to compute the ASDU bytes ($X_{d}$), the APCI bytes ($X_{h}$), and $(f_{ASDU},f_{APCI})\in f_{APDU}$. In the case where a number of fragments are required in the transmission, then $f_{APDU}\in f_{{APDU}_{n}}$ and *n* = 1, 2, 3, …, k, where k is the limited integer. Moreover, the computed bytes are simultaneously updated by the employment of function $f_{\mathrm{dy}}$ and the designation of $X_{dy}$. The computed fragment (Frag) with the concatenation (||) of dynamic bytes can be demonstrated as

$$\begin{array}{c} \forall X_{\alpha \in \left(S;R\right)}\Rightarrow \text{Frag}=f_{APDU}\left(X\right)=f_{APCI}\left(X\right)+\;f_{ASDU}\left(X\right)||X_{\begin{array}{c} dy \end{array}},\\ f_{APCI}\left(X\right)\Rightarrow X_{\begin{array}{c} h \end{array}}\wedge f_{ASDU}\left(X\right)\Rightarrow X_{\begin{array}{c} d \end{array}}\wedge (X_{\begin{array}{c} h \end{array}},X_{\begin{array}{c} d \end{array}})\leq Limit \end{array}$$

where limit is a maximum value depending on the size of the bytes that are specified for a fragment.

*Postulate 2*.  The existent functions $f_{AC},f_{FC}$, and $f_{IIN}$, are employed to compute the protocol-header bytes, if, and only if, the parameters $x_{1},x_{2},x_{3}x_{4},x_{\left(im,ex\right)},\mathrm{and}x_{iin}$ are satisfied, such that $f_{AC},f_{FC},f_{IIN}\in f_{APCI}$.

In the case of sending fragment, $\exists X_{\begin{array}{c} h \end{array}}\Leftrightarrow (x_{\begin{array}{c} 1 \end{array}},x_{\begin{array}{c} 2 \end{array}},x_{\begin{array}{c} 3 \end{array}}x_{\begin{array}{c} 4 \end{array}})\;\in f_{AC}\wedge \left(x_{\begin{array}{c} \left(im,ex\right) \end{array}}\right)\in f_{FC}\;\wedge \left(f_{AC},f_{FC}\right)\in \;f_{APCI}$.

In the case of a response fragment,

$$\exists X_{\begin{array}{c} h \end{array}}\Leftrightarrow (x_{\begin{array}{c} 1 \end{array}},x_{\begin{array}{c} 2 \end{array}},x_{\begin{array}{c} 3 \end{array}}x_{\begin{array}{c} 4 \end{array}})\;\in f_{AC}\wedge \left(x_{\begin{array}{c} \left(im,ex\right) \end{array}}\right)\in f_{FC}\;\wedge \left(x_{\begin{array}{c} iin \end{array}}\right)\in f_{IIN}\wedge (f_{AC},f_{FC},f_{IIN})\in \;f_{APCI}$$

To compute the header (h) bytes, the AC function $f_{AC}$ and its parameters are as follows: $\left[x_{1},x_{2},x_{3}x_{4}]==[\mathrm{Sequence}\mathrm{Number},\mathrm{Confirmation},\mathrm{FIN},\mathrm{FIR}\right]$. The function $f_{FC}$ with its parameter-request code (im) and/or response code (ex) are performed in the case of sending (S) a fragment, while the difference of the two-byte IIN is counted via the function $f_{IIN}$ during the responding (R) fragment.

*Postulate 3*.  The protocol-data bytes are computed using the employment of the functions $f_{OH}\mathrm{and}f_{OD}\in f_{ASDU}$, if, and only if, the parameters $\left(x_{5},x_{6},x_{7},\mathrm{and}x_{8}\right)$ are satisfied; a difference of two bytes exists in the response case.

$$\exists X_{\begin{array}{c} d \end{array}}\Leftrightarrow [(x_{\begin{array}{c} 5 \end{array}}∋x_{\begin{array}{c} 5.1 \end{array}},x_{\begin{array}{c} 5.2 \end{array}}),(x_{\begin{array}{c} 6 \end{array}}∋x_{\begin{array}{c} 6.1 \end{array}},x_{\begin{array}{c} 6.2 \end{array}}),x_{\begin{array}{c} 7 \end{array}}]\in f_{OH}\wedge \left(x_{\begin{array}{c} 8 \end{array}}\right)\in f_{OD}\;\wedge \left(\;f_{OH},f_{DO}\right)\in \;f_{ASDU}$$

For the ASDU bytes, the object header (OH) function $f_{OH}$ with its parameters$\left[x_{5},x_{6},x_{7}]==[\mathrm{Object}\mathrm{Function}(\mathrm{of}),\mathrm{Object}\mathrm{Qualifier}(\mathrm{oq}),\mathrm{Object}\mathrm{Range}(\mathrm{or})\right]$, and the data object (DO) function $f_{DO}$ with its parameter [$x_{8}$] == [user bytes (ub)] are computed in the case of sending (S)/responding (R), but a two-byte difference is accounted for in the responding (R) case that ensures the total size. Here, $\left[x_{5.1},x_{5.2}]==[\mathrm{Object}\mathrm{Group}(\mathrm{og}),\mathrm{Object}\mathrm{Variation}(\mathrm{ov})\right]$ are implied for the object function (of) and $\left[x_{6.1},x_{6.2}]==[\mathrm{Qualifier}\mathrm{code}(\mathrm{qc}),\mathrm{Index}\mathrm{Size}(\mathrm{is})\right]$ are implied for the object qualifier (oq).

*Postulate 4*.  The function $f_{L}$ is an explicit, dual logical-distribution function if it is operated on bytes X, and X are retrieved from function $f_{\mu }$, as follows:

$$\;f_{\mu }\Rightarrow \forall X_{\alpha \in \left(S;R\right)}\Rightarrow f_{ASDU}\left(X\right)\Rightarrow X_{\begin{array}{c} d \end{array}}\Rightarrow f_{L}\left(X_{\begin{array}{c} d \end{array}}\right)\Rightarrow f_{L}\left(X_{\begin{array}{c} d \end{array}}\right)\approx \sum_{i=1}^{r}{\left(DB\right)}_{i},i=1,2,3,\ldots r$$

here, the ASDU bytes are logically (L) fixed into the data blocks (DB) but the bytes vary in the cases of sending and responding, and r is represented for the logical 8 blocks in the case of a full fragment (*i.e.*, 1992 bytes and $f_{ASDU}\in f_{APDU}$).

## 7. Algorithm: Pseudo-Code Computing of Fragment with Security

**Algorithm 1** Fragment Security**Input:** User layer bytes $X,X_{\begin{array}{c} \alpha \end{array}}$,α∈(S;R);**Output:** ($X_{\begin{array}{c} h \end{array}},X_{\begin{array}{c} d \end{array}})$, Secu rity(Encryption Eny, Decryption Dny, Messag(M), $X_{dy}$Define and manage the number of user layer bytes , $X,X_{\begin{array}{c} \alpha \end{array}}$,α∈(S;R);;Calculate$\;X_{\begin{array}{c} h \end{array}}=\{(AC,\;FC)|(x_{\begin{array}{c} 1 \end{array}},x_{\begin{array}{c} 2 \end{array}},x_{\begin{array}{c} 3 \end{array}}x_{\begin{array}{c} 4 \end{array}},x_{\begin{array}{c} \left(im,ex\right) \end{array}})\;\leq limit,\;limit\in S\};$$\;X_{\begin{array}{c} h \end{array}}=\left\{\left(AC,\;FC,IIN\right)|\left(\;x_{\begin{array}{c} 1 \end{array}},x_{\begin{array}{c} 2 \end{array}},x_{\begin{array}{c} 3 \end{array}}x_{\begin{array}{c} 4 \end{array}},x_{\begin{array}{c} \left(im,ex\right) \end{array}},x_{\begin{array}{c} iin \end{array}}\right)\leq limit,\;limit\in R\right\};$Calculate $\;X_{\begin{array}{c} d \end{array}}=\{\left(OH,\;OD\right)|(\;x_{\begin{array}{c} 5 \end{array}},x_{\begin{array}{c} 6 \end{array}},x_{\begin{array}{c} 7 \end{array}}x_{\begin{array}{c} 8 \end{array}})\leq limit,\;limit\in \text{(S; R)}\};\approx {\left(DB\right)}_{i},1\leq i\leq 8;$By usage of steps 2 and 3, $\text{APDU}=\{\left(X_{\begin{array}{c} h \end{array}},X_{\begin{array}{c} d \end{array}}\right)|\sum^{}\left(X_{\begin{array}{c} h \end{array}},X_{\begin{array}{c} d \end{array}}\right),\text{limit}<\sum^{}\left(X_{\begin{array}{c} h \end{array}},X_{\begin{array}{c} d \end{array}}\right)\leq \text{limit\}},\alpha \in \left(S;R\right);$ $X_{\begin{array}{c} dy \end{array}}=dy.Update\left(\;\right)$;While there is application protocol data unit (APDU) computed bytes ($\;X_{\begin{array}{c} h \end{array}},X_{\begin{array}{c} d \end{array}})$,α∈(S;R);Security is computed by employing of cryptography functions, Symmetric (Sym)function, Asymmetric (Aym) function, Hashing (H) function; Eny (APDU)==Messag(M)==$\text{Eny}\left[\text{Sym}\left(\text{APDU }\right),\text{Aym}\left\{\text{Hash}\left(\text{APDU}\right)\right\}\right];\;X_{\begin{array}{c} dy \end{array}}=dy.Update(\;$);If Eny (APDU) bytes are received at target side. Then$\text{ Dny}\left[M\right]==\text{Dny}\left[\text{Eny}\left\{\text{Sym}\left(\text{APDU }\right),\text{Aym}\left\{\text{Hash}\left(\text{APDU }\right)\right\}\right\}\right];X_{\begin{array}{c} dy \end{array}}=dy.Update\left(\;\right);$End ifIf Dny[M] is performed. Then($\;X_{\begin{array}{c} h \end{array}},X_{\begin{array}{c} d \end{array}})$ are reassemble to bytes X;End ifEnd while

## 8. Implementation

The aim of this work is to design the DNP3 stack and to deploy the security algorithm(s) within the protocol; therefore, the DNP3 stack has been designed by the deployment of explicit (or real) functions in the C# programming platform, while the historian (or database) was deployed by using the MySQL tool. Several approaches [1,3,5,6,7,14,15] were analyzed against the SCADA/DNP3 in terms of security but they are based on end-to-end developments. DNP3 is a proprietary protocol and is situated at the top of the TCP/IP protocols for Internet-based communication [5,8,9,14,15,57,58,59,60]. Security is a major issue that has been faced by the DNP3 protocol since Internet-based communication has occurred through open protocol(s). This work therefore proposes a new approach that deploys the security within the DNP3 protocol before the transmission of bytes to non-proprietary protocols such as TCP/IP and UDP.

In the implementation phase, the design of the DNP3 stack is also a major challenge, and the deployment of security using a strong mechanism that has the potential to secure the SCADA/DNP3 transmission against attacks is required. The first challenge was resolved by the design of the DNP3 stack and its relevant functions in C# using implicit/explicit libraries and functions. Prior to the security-implementation phase, another major challenge is the storage, updating, and maintenance of the security tracks with the corresponding stack information during the real-time delivery of bytes. To resolve this challenge, the DCB is deployed and employed during the entire development stage. The DCB contains the numbers of the dynamic functional fields that successfully control the security operations and stack relevant information.

### 8.1. Dynamic Cryptograph Buffer

**Figure 6:**
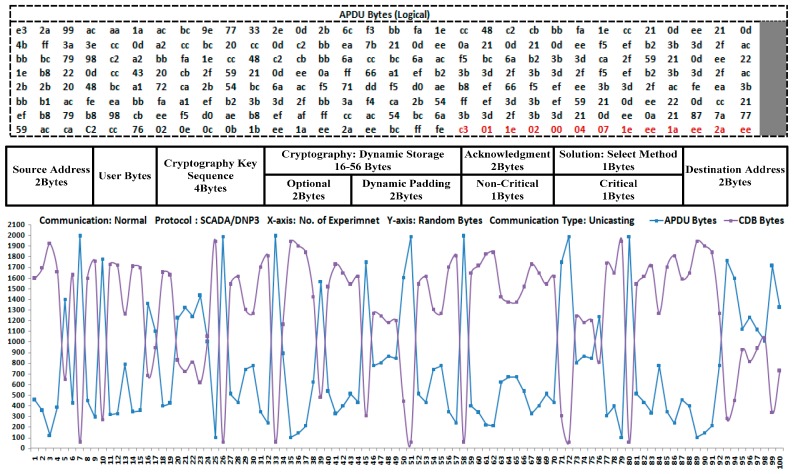
APDU bytes flow with the DCB interaction: At the initial stage, the number of bytes is manipulated within the application-layer stack as a part of the DNP3 protocol. The black-color bytes are designated for user-manipulated bytes, while the red-color bytes c3: Application Control (AC), 01: Function Code (FC), 1e: Group, 02: Variation, 00: Qualifer, 04: Start, 07: Stop are operational, specified bytes of the application layer, and the remaining bytes such as 1e, ee, 1a, ee, 2a, and ee are security-implementation bytes. During implementation, the performances-measure lines in this figure show the overall byte flow within the stack, and the dynamic allocation of the DCB bytes depends on the APDU bytes.

During the security-development phase, the DCB was employed to store the corresponding information of the security implementation and the flow of bytes within the stack. In Figure 6, the original DCB size is 56 bytes but this is likely to be dynamic due to the APDU-manipulated bytes. The DCB contains the number of functional fields with a variety of storage sizes that have been employed to keep the security tracks of the implementation and other related stack information. The performance results evaluated that the original DCB space (56 bytes) is sufficient for overall information storage, even in a case where the application stack is full or corresponds to the maximum number of bytes.

The bytes have been encrypted and the corresponding information is updated within the DCB. The “user bytes field” in the DCB is a dynamic storage field that specifies the byte sizes (or the APDU bytes) that are being employed after the development of the security process. The additional space is dynamically allocated to the “cryptography: dynamic storage” field in the DCB by the operation of the “byte-shifting function”. The initial size of the “cryptography: dynamic storage” is 16__56 in DCB, but this would be increased by the indication from the “user bytes field”; subsequently, if these bytes are not required by the DCB, then these are treated as padding bytes (or shifted to “padding field”).

This operation also indicates that the overall message is constructed and prepared for transmission while the message contents are verified by an operational field called “Optional”. A two-byte (unassigned) acknowledgment field has been employed to monitor the delivery of a message during transmission. This field contains two flags such as ACK: flag (0): bytes delivery without acknowledgment, and ACK: flag (1): bytes delivery with acknowledgment.

During security implementation, the following three types of algorithms were employed: symmetric AES algorithm, asymmetric RSA algorithm, and SHA-2 hashing algorithm. The corresponding keys are generated, deployed, and added in distinct sequence numbers. Each time that a message has been encrypted/decrypted, the counter is incremented and the value is added in the “Cryptography Key Sequence” field. Two explicit flags such as flag (0): Security__Fail and flag (1): Security__Pass were also employed to monitor the status of the security development. Two especial fields are used in the DCB and are designated as Critical (bytes) and Non-Critical (bytes), these are observed to report the transmission status during abnormal/normal scenarios.

The request and response headers are distinct and are defined at the application layer, and the corresponding addresses, such as the source and destination addresses, are defined in the link header or link-protocol-control information (LPCI) at the data-link layer as a part of the DNP3 protocol. In this study, security is deployed on the APDU bytes in a case where the APCI is not encrypted, so there are many chances of attacks, such as header modification and alters, header reply, header deletion, and/or other integrity and authentication attacks [56,57,59].

To resolve this issue, four bytes are used from “cryptography: dynamic storage” as a part of the DCB; in cases where the request or response APCI is not readable at the lower layers and/or at the target side due to encryption, the designated four bytes are employed to define the external application-layer header (or APCI) that travels along with the encrypted message (or APDU). This approach is significant when the adversary successfully modifies and/or alters the APCI information (or external APCI information) because, at the target side, the external APCI information will be verified after a comparison with the encrypted APCI as a part of APDU encryption process. This approach would also be significant and provides the future security directions for the data-link layer of the DNP3 protocol [5,59]; in this case, the LPCI will be encrypted and required to define the external addresses, and the allocated four bytes of the source address and destination address (fields) in the DCB would be used for that purpose.

According to the SCADA/DNP3 transmission requirements, a variety of cryptography methods that do not interfere with the communication process have been analyzed to achieve enhanced security results. A cryptography key method has been deployed to save the session during the encryption/decryption process. In this method, the user bytes (or APDU bytes) are not encrypted, and only the key is appended with the desired bytes that minimize the computation time during a security development. The following function shows the encryption/decryption of protocol bytes by using the key-appended method (AP):

$$f_{Sym}\Rightarrow Eny_{\left(Sym\right)}\left[AP.SC_{\begin{array}{c} k_{S} \end{array}}\begin{array}{c} \left\{\text{Frag}\right\} \end{array}\right]$$

where the symmetric (Sym) function ($f_{Sym}$) is employed, and the secret key $SC_{\begin{array}{c} k_{S} \end{array}}$ is appended (AP) with the fragment (Frag).

A field designated “Solution: Select Method” has been employed within the DCB and reports the security method(s) and the relevant algorithms being used for the secure communication process. Based on SCADA/DNP3 communications including unicasting, multicasting, and broadcasting and their requirements, the security is best designed, deployed, and reported by the “Solution: Select Method” field. This functional field dynamically selects the security solution(s) according to the requirements of a SCADA/DNP3 communication; for example, in multicasting and broadcasting, the asymmetric algorithms are not appropriated because of the number of keys that are required with heavy session(s) during security deployments; therefore, symmetry-based cryptography solutions are convenient for these kinds of transmissions.

### 8.2. Cryptograph

In the SCADA/DNP3 testbed, the historian shares among the configured nodes (or MTU/RTU) via the secure channels that are established between them. The historian has a number of fields including the “MTU public key”, “RTU public key”, “shared secret key”, “MTU/RTU IP addresses”, and “logical port numbers”. Cryptography keys are generated and employed in the desired fields with identifiers so that K→0x0001,0x0002,….., k−1 and, further, would be deployed during encryption/decryption processes.

The SCADA/DNP3 protocol contains the relevant functions including “read”, write”, select”, and “operate” with the specified codes [8,9,52]. The security implementation (using a cryptography mechanism) is two-fold: the security has been deployed in correspondence to each protocol function, and computed bytes are transmitted from the application layer of the DNP3 protocol.

Example 1: Suppose that read/write functions are being performed by an application layer. S.t.

(Read; Write): AC == (c3; c3), FC == (01, 02), Group G == (ie; 32), Variation V == (02, 01), Qualifier Q(00, 07), Start == (04, 01), Stop == (07, 01), Encryption E == (1e, 1e).

The security was deployed and reacted as an additional layer that is designated as a security layer for testing the APDU bytes; that is, the APDU bytes are constructed and encrypted at the security layer and upon the receipt of the bytes, a decryption process can be performed at that layer. This security layer acts as a safeguard for APDU bytes, and this implementation has been selected as a strong security development against the application layer attacks as a part of the DNP3 protocol. The details are related to the following of the encryption and decryption processes and, to be convenient, the basic notations that were used during the encryption/decryption processes are summarized in Table 2.

**Table 2:** Terminologies for security development.

Notations	Descriptions
$f_{Eny}\left(Frag\right)$ **	*Encryption (Eny) function* $f_{Eny}$ *of fragment* ( Frag) *or APDU bytes.*
$f_{Sym};f_{Aym};\;f_{H}\in f_{Hy}$ **	*Security-development functions: Symmetric (* $f_{Sym}$ *), Asymmetric (* $f_{Aym}$ *), and Hashing (* $f_{H}$ *), and combined as hybrid (Hy) function during encryption/decryption process.*
$SC_{\begin{array}{c} k_{S} \end{array}},\;Pr_{\begin{array}{c} k_{S} \end{array}},\;Pu_{\begin{array}{c} k_{T} \end{array}},\;H_{S}$ **	*Shared Secret Key (* $SC_{\begin{array}{c} k_{S} \end{array}}$ *) used by sender (S), Sender Private Key (* $Pr_{\begin{array}{c} k_{S} \end{array}}$ *), Target (T) or Receiver Public Key (* $Pu_{\begin{array}{c} k_{T} \end{array}}$ *), and Sender (S) Hashing (* $H_{S}$ *).*
*(* $M_{1}$ *,* $M_{2},M_{3}$ *)* ∈M	*Computed values after deployment of encryption* $Eny{\;}_{\left(Sym;Aym;H\right)\;}\left(b,k_{S}\right)$ *, with bytes (b) and* $k_{S}\;$ *is designated for number of sender (S) keys (k) that are applied during encryption, and are member of Message (M)*
*(* $W_{\begin{array}{c} 1 \end{array}},\;W_{\begin{array}{c} 2 \end{array}},\;W_{\begin{array}{c} 3 \end{array}})\in W$ **	*(* $W_{\begin{array}{c} 1 \end{array}},\;W_{\begin{array}{c} 2 \end{array}},\;W_{\begin{array}{c} 3 \end{array}})\;are$ *initialized and show the work done after the encryption process, and they are members of Work done (W).*
Tpy **	$Total_{Payload}\left(Tpy\right)$ *or complete payload after encryption process.*
$f_{Dny}(\;Tpy)$ **	*Decryption (Dny) function* $f_{Dny}$ *of* Total_Payload (Tpy) *.*
$f_{CP},f_{CP}\left(Val\right)$ **	*Hash comparison* (CP) *function* $f_{CP}$ *and computed hash values (val) as* $f_{CP}\;\left(Val\right).$ **
$SC_{\begin{array}{c} k_{T} \end{array}},\;Pr_{\begin{array}{c} k_{T} \end{array}},\;Pu_{\begin{array}{c} k_{S} \end{array}},\;H_{T}$ **	*Shared Secret Key (* $SC_{\begin{array}{c} k_{T} \end{array}}$ *) used at target (T) side, target (T) Private Key (* $Pr_{\begin{array}{c} k_{T} \end{array}}$ *), Sender (S) Public Key (* $Pu_{\begin{array}{c} k_{S} \end{array}}$ *), and Target (T) Hashing (* $H_{T}$ *).*
$f_{Ass}$ **	*Assemble (* Ass *)* *function* $f_{Ass}$ *is used to assemble the constructed application-layer bytes* $\left(X_{\begin{array}{c} h \end{array}},X_{\begin{array}{c} d \end{array}}\right)$ *to the lower-layer bytes* (Y) *.*
$f_{Rss}$ **	*Reassemble (* Rss *)* *function* $\;f_{Rss}\;$ *is used to reassemble the protocol bytes (* $X_{\begin{array}{c} h \end{array}},X_{\begin{array}{c} d \end{array}})\;$ *with the user-layer bytes* X. **

*Encryption 1*:  MTU uses a secret key from the historian that is generated by using the AES algorithm and also encrypts the APDU bytes using this key $\approx W_{\begin{array}{c} 1 \end{array}}$. The APDU bytes or ${\left(\text{APDU}\right)}_{n}$ are then subjected to the SHA-2 hashing algorithm for an integrity value check; therefore, ${\left(\text{APDU}\right)}_{n}$ uses the input for the SHA-2 hashing algorithm to generate a hash value; it is also represented as ${\left(\text{APDU}\right)}_{n}$ digest or ${\left(\text{APDU}\right)}_{n}$_DIGEST. When the hashing process is complete, the SHA-2 hashing algorithm followed by the ${\left(\text{APDU}\right)}_{n}$_DIGEST is encrypted with the private key$\;\approx W_{\begin{array}{c} 2 \end{array}}$ that was generated from the RSA algorithm. This process produces a digital signature to verify the non-repudiation security (service).

**Figure 7:**
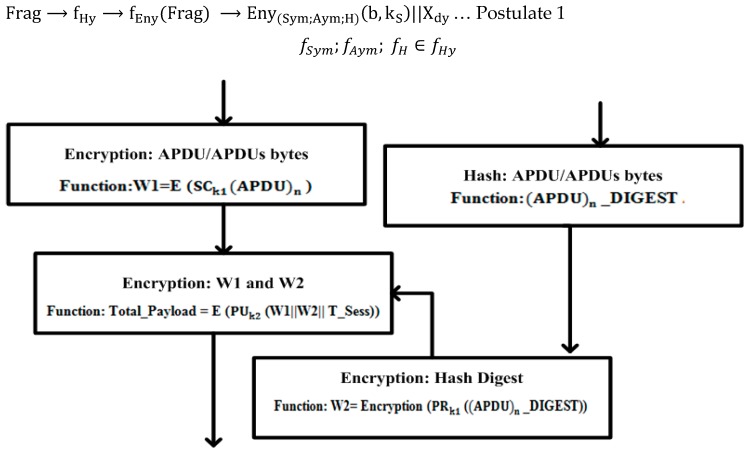
Encryption process: application-layer implementation.

After this, the functions W1 and W2 are concatenated with each other and are then encrypted again with a public key of RTU, using the RSA algorithm. The completion of this process produces the new function “Total_Payload”. The encryption information is updated in the DCB and Figure 7 shows the cryptography implementation during the encryption process.

*Postulate 5*.  The function$\;f_{Eny}$ is a combination of three security functions ($f_{Sym};f_{Aym};\;f_{H})\;$whereby $f_{Eny}$ can be computed with the hybrid function $f_{Hy}$ and some computed values ($M_{1}$,$M_{2},M_{3}$) ∈M, ($W_{\begin{array}{c} 1 \end{array}},W_{\begin{array}{c} 2 \end{array}},\;W_{\begin{array}{c} 3 \end{array}})\in W$, and M≈W. The mapping function $f_{{\left(MP\right)}_{\begin{array}{c} 1 \end{array}}}$: $\text{Frag}\to f_{Eny}\left(\mathrm{Frag}\right)\Rightarrow$

$\text{Frag}\to f_{\text{Hy}}\to f_{\text{Eny}}\left(\text{Frag}\right)\to {\text{Eny}}_{\left(\text{Sym};\text{Aym};H\right)}\left(b,k_{S}\right)||X_{\begin{array}{c} dy \end{array}}$… Postulate 1

$$f_{Sym};f_{Aym};\;f_{H}\in f_{Hy}$$

$Eny_{\left(Sym;Aym;H\right)}\left(b,k_{S}\right)\;$shows the number of bytes (b) in a fragment (Frag) being manipulated by the hybrid function $f_{Hy}$, where $k_{S}\;$is designated for the number of sender (S) keys (k) that are applied during encryption (Eny).

$$\underset{Eny}{\Leftrightarrow }\begin{array}{c} f_{Sym}\Rightarrow Eny_{\left(Sym\right)}\left[SC_{\begin{array}{c} k_{S} \end{array}}\begin{array}{c} \left\{\text{Frag}\right\} \end{array}\right]\Rightarrow M_{1},\; \end{array}M_{1}\in M,\;f_{Sym}\in f_{Hy}$$

The symmetric encryption function $f_{Sym}$ is deployed on the APDU bytes including the number of fragments ${\left(APDU\right)}_{n}$ that have been received and manipulated with encryption. To distinguish a successful encryption operation from the others, $W_{\begin{array}{c} 1 \end{array}}\approx M_{1}$ is initialized and shows the successful work that is completed after an encryption process.

$$\underset{Eny}{\Leftrightarrow }\begin{array}{c} \left(f_{Aym};\;f_{H}\right)\Rightarrow Eny_{\left(Aym;H\right)}\left[Pr_{\begin{array}{c} k_{S} \end{array}}\left\{H_{S}\left(\text{Frag}\right)\right\}\right]\Rightarrow M_{2},M_{2}\in M, \end{array}\left(f_{Aym};\;f_{H}\right)\in f_{Hy}$$

The digital signature is formed by the encryption of the hash-digest bytes, and W_2_ $\approx M_{2}$ is designated for the work done after the completion of the encryption process via the asymmetric (Aym) function $f_{Aym}$ and the hashing (H) function $f_{H}$. The hashing function $f_{\;H}\;$is deployed first and the digital signature is then produced after the deployment of the sender (S)′sprivate key $Pr_{\begin{array}{c} k_{S} \end{array}}$ on hash value, as follows:

$$\underset{Eny}{\Leftrightarrow }\begin{array}{c} f_{Aym}\Rightarrow Eny_{\left(Aym\right)}\left[Pu_{\begin{array}{c} k_{T} \end{array}}\left(M_{2}||M_{1}\right)\right]\Rightarrow M_{3},\;M_{3}\in M, \end{array}f_{Aym}\in f_{Hy}$$

The message$\;\left(M_{3}\right)$ is computed by the employment of the target (T) public key $Pu_{\begin{array}{c} k_{T} \end{array}}$ on $M_{1}$ and $M_{2}$ via the asymmetric-encryption function $f_{Aym}$, so that ($M_{1}$,$M_{2},M_{3}$) ∈M, ($W_{\begin{array}{c} 1 \end{array}},W_{\begin{array}{c} 2 \end{array}},\;W_{\begin{array}{c} 3 \end{array}})\in W$, and $M\approx W=={\text{Total}}_{\text{Payload}\left(Tpy\right)}==Eny_{\left(Aym\right)}\left[Pu_{\begin{array}{c} k_{T} \end{array}}\left(W_{2}||W_{1}\right)\right],\text{and the payload is }$complete after the encryption process.

*Decryption 1*:  The RTU receives a Total_Payload from the lower layer of the DNP3 protocol and before a conversion to the application-layer fragment or APDU bytes, Total_Payload is treated first with the cryptography solution for the purposes of the decryption process. The RTU uses a private key that is generated by the RSA algorithm and decrypts the Total_Payload. Here, the generated function W1 is subjected to decryption by using the AES algorithm. The MTU and RTU have been sharing the secret key via a secure channel that has been established between them; therefore, the function W1 is decrypted successfully before the APDU bytes are reformed.

When the whole decryption process has been completed successfully and the APDU bytes are reformed, the APDU bytes are then subjected to the SHA-2 algorithm for an integrity value check; therefore, the APDU bytes are used as an input for the SHA-2 hash algorithm and generate a hash digest.

**Figure 8:**
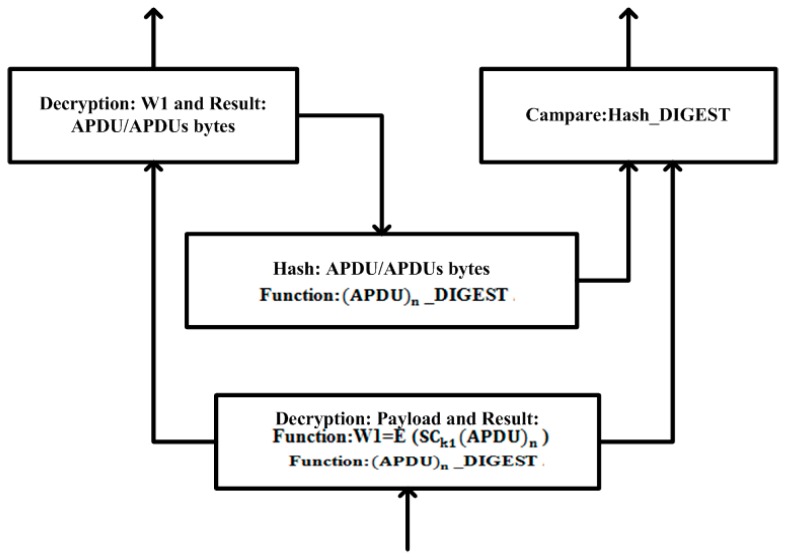
Decryption process: application-layer implementation.

When the MTU/RTU hash-digest values have been matched successfully, the APDU bytes are then accepted and the integrity of the APDU bytes is also verified. Otherwise, the APDU bytes are rejected in the application layer on the RTU side and an acknowledgment will be sent from the RTU to the MTU to correct the APDU bytes, or to raise an alert regarding an attack. Figure 8 illustrates the cryptography implementation during the byte-decryption process.

*Postulate 6*.  The function $f_{Hy}$ is a hybrid function if it is computed by the function$\;f_{Dny}$: $f_{Dny}\to$ ($f_{Sym};f_{Aym};\;f_{H})$, while some computed values of Tpy exist and the corresponding functions are reciprocal of$\;f_{Dny}$. Suppose $f_{Eny}$ is an encryption function and its computed value is decrypted by function $f_{Dny}.$ The mapping function is$f_{{\left(MP\right)}_{\begin{array}{c} 2 \end{array}}}$: $f_{Eny}\left(\text{Frag}\right)\to f_{Dny}\left[f_{Eny}\left(\text{Frag}\right)\right]\Rightarrow$ $Tpy\to f_{Hy}\to f_{Dny}(Tpy)\to Dny_{\left(Sym;Aym;H\right)}(Tpy,\;k_{T})\to \text{Frag }||X_{\begin{array}{c} dy \end{array}}$:

$$f_{Sym};f_{Aym};\;f_{H}\in f_{Hy}$$

$\underset{Dny}{\Leftrightarrow }f_{Dny}(Tpy)$ = =$f_{Dny}\left[Eny_{\left(f_{Aym}\right)}\{Pu_{\begin{array}{c} k_{R} \end{array}}\left(W_{2}||W_{1}\right)\}\right]$ when the Sender (S) encrypted bytes have been received and the decryption process is being performed.

$\Rightarrow Dny_{\left(Aym\right)}\left[\left(Pu_{k_{S}};Pr_{\begin{array}{c} k_{T} \end{array}}\right)\begin{array}{c} \left\{Tpy\right\} \end{array}\right]$ is the decryption process that is performed using the Sender(S) public key$\;Pu_{k_{S}}$and the target (T)private key $Pr_{\begin{array}{c} k_{T} \end{array}}$, via an asymmetric algorithm. As a consequence, we get the sender (S) hash value ${\left(H\_Digest\right)}_{S}\;$of the fragment and the encrypted value$\;W_{1}\;$via a shared secret key (*i.e.*,$\;SC_{\begin{array}{c} k_{S} \end{array}}==SC_{\begin{array}{c} k_{T} \end{array}}).$

$\Rightarrow Dny_{\left(Sym\right)}\left[SC_{k_{T}}\begin{array}{c} \left\{W_{1}\right\} \end{array}\right]\Rightarrow \text{ Frag}$ is the decryption process that is performed using the target (T)secret key $\left(SC_{\begin{array}{c} k_{T} \end{array}}\right)$from the symmetric algorithm. As a consequence, the following fragment is obtained:

$$\begin{array}{c} \Rightarrow f_{Hy}\to f_{H}\to H_{T}[{\left(Frag\right)}_{S}]\to {\left(H\_Digest\right)}_{T}\\ \Rightarrow f_{CP}[{\left(H\_Digest\right)}_{S\;},\left(H\_Digest{)}_{T}\right]\Rightarrow f_{CP}\left(Val\right).\{\begin{array}{c} Equal\;\\ Not\;Equal\; \end{array} \end{array}$$

The hash value is calculated at the target (T)side by$\text{byt}.H_{T}[{\left(\text{Frag}\right)}_{S}]\to {\left(H\_\text{Digest}\right)}_{T}$, and the value is then compared with the sender (S) hash value ${\left(H\_Digest\right)}_{S}$, using a comparison of the (CP) function $f_{CP}$. Such is the $f_{CP}\left(Val\right)$ that if the computed hash values (val) are equal then the bytes are accepted; otherwise, these are rejected with the reply message “unauthorized bytes”.

After the decryption process, the APDU bytes that would be (further) used by the user-application layer are reassembled. To validate the APDU reassembling process at the receiver side, Postulate 7 is employed, while Postulate 8 validates the overall proposed security design and development. To be convenient, the basic notations of Postulate 7 are summarized in Table 2, while the notations used for Postulate 8 are summarized in Table 1 to Table 2.

*Postulate 7*.  Suppose the protocol bytes are assembled according to the use of the assembled function $f_{Ass}:f_{Ass}\to Y,$ then the reassemble function $f_{Rss}:f_{Rss}\to X$ reassembles the protocol bytes ($X_{\begin{array}{c} h \end{array}},X_{\begin{array}{c} d \end{array}})\;$with user-layer bytes X. The mapping function is represented by $f_{{\left(MP\right)}_{\begin{array}{c} 3 \end{array}}}$: $f_{APDU}\left(X\right)\to \left[f_{Rss}\left(X_{\begin{array}{c} \left(h,d\right) \end{array}}\right)\right]\to X$.

$$\Rightarrow \text{Frag}=f_{APDU}\left(X\right)\to (X_{\begin{array}{c} h \end{array}},X_{\begin{array}{c} d \end{array}})\to \left[f_{Rss}\left(X_{\begin{array}{c} \left(h,d\right) \end{array}}\right)\right]\to X$$

After the decryption process of Postulate 6, the function$\;f_{Rss}\;$is employed to reassemble the protocol bytes ($X_{\begin{array}{c} h \end{array}},X_{\begin{array}{c} d \end{array}})$ with the user-application layer bytes X, as follows:

$$\begin{array}{l} \Rightarrow \{f_{APCI}\left(X\right),f_{ASDU}\left(X\right)\}\in f_{APDU}\left(X\right)\;\vee f_{APCI}\left(X\right)\in f_{APDU}\left(X\right)\wedge f_{ASDU}\left(X\right)\in f_{APDU}\left(X\right)\\ \Rightarrow \{f_{APCI}\left(X\right),f_{ASDU}\left(X\right)\}\to (X_{\begin{array}{c} h \end{array}},X_{\begin{array}{c} d \end{array}})\vee f_{APCI}\left(X\right)\to X_{\begin{array}{c} h \end{array}}\;\wedge f_{ASDU}\left(X\right)\to X_{\begin{array}{c} d \end{array}} \end{array}$$

Then, the function $f_{Rss}$ reassembles with the interaction of the bytes $X_{\begin{array}{c} \left(h,d\right) \end{array}}$and $f_{Rss}\left(X_{\begin{array}{c} \left(h,d\right) \end{array}}\right)$: $X_{\begin{array}{c} h \end{array}}\left[f_{Rss}\left(X_{\begin{array}{c} \left(h,d\right) \end{array}}\right)\right]\;X_{\begin{array}{c} d \end{array}}$. Here, the header bytes $(X_{\begin{array}{c} h \end{array}})\;$are split from the ASDU bytes ($\;X_{\begin{array}{c} d \end{array}})$ and the user-application-layer bytes are reformed; however, the assemble function $f_{Ass}:f_{Ass}\to Y,$ is used to assemble the constructed application-layer bytes $\left(X_{\begin{array}{c} h \end{array}},X_{\begin{array}{c} d \end{array}}\right)$ with the lower-layer bytes (Y).

*Postulate 8*.  The function $f_{\mu }$defines the number of bytes X from the user layer, and these bytes are constructed ($X_{\begin{array}{c} h \end{array}},X_{\begin{array}{c} d \end{array}},\text{ dynmaicbytes }X_{\begin{array}{c} dy \end{array}},\text{ identifier}$ α) and manipulated using the security function$\;f_{Hy}$: $f_{Eny/Dny}\to$ ($f_{Sym};f_{Aym};\;f_{H}).$

$\forall X_{\alpha \in \left(S;R\right)}$ are “n” bytes of information (with the appropriate function code and data object) that are passed from the user-application layer, such that:

$\forall X_{\alpha \in \left(S;R\right)}\Rightarrow \text{Frag}=f_{APDU}\left(X\right)=f_{APCI}\left(X\right)+f_{ASDU}\left(X\right)||X_{\begin{array}{c} dy \end{array}}$…… Postulate 1

$\Leftrightarrow (f_{AC},f_{FC},f_{IIN})\in f_{APCI}\Rightarrow f_{APCI}\left(X\right)\Rightarrow X_{\begin{array}{c} h \end{array}}\wedge (f_{OH},f_{OD})\in f_{ASDU}\Rightarrow f_{ASDU}\left(X\right)\Rightarrow X_{\begin{array}{c} d \end{array}}$…… Postulates 2 and 3.

The bytes X are retrieved from the user-application layer, and the further function $f_{APDU}\left(X\right)\;$is deployed to compute the application-layer fragment (Frag) with the concatenation (||) of the dynamic bytes $X_{\begin{array}{c} dy \end{array}}$. Moreover, the function $f_{APCI}\left(X\right)$ is employed for the APCI bytes ($X_{\begin{array}{c} h \end{array}}$) and the function $f_{ASDU}\left(X\right)$ is employed for the ASDU bytes ($X_{\begin{array}{c} d \end{array}}$) in the case of sending (S)/responding (R) α∈(S, R), as follows:

$$\Rightarrow f_{APDU}\left(X\right)\Rightarrow f_{APDU}\left(X\right).Val\{\begin{array}{c} \leq Limit\\ \geq Limit\\ ==0\; \end{array}$$

A limit designates the number of bytes that can be computed from $[f_{APCI}\left(X\right),f_{ASDU}\left(X\right)],$ and in the case of “0”, only $f_{APCI}$ is executed. These bytes are updated in correspondence to the dynamic buffer $X_{\begin{array}{c} dy \end{array}}$ using the function$\;f_{dy},\;\text{as follows}:$

$\Rightarrow f_{Hy}$: $f_{Eny}\to$ ($f_{Sym};f_{Aym};f_{H})\left[f_{APDU}\left(X\right)\right]||f_{dy}$ …… Postulates 1 and5

$\Rightarrow f_{Hy}$: $f_{Dny}\to$ ($f_{Sym};f_{Aym};f_{H})\left[Tpy\right]||f_{dy}$ …… Postulates 5 and 6

The encryption function $f_{Eny}$ of $\left[f_{APDU}\left(X\right)\right]$ and the decryption function $f_{Dny}$ of [Tpy] are computed, and the corresponding values are updated within the dynamic buffer using function $f_{dy}$, as follows:

$$\Rightarrow f_{dy}\left(X\right).\text{Val}\{\begin{array}{c} \leq Limit\\ \geq Limit\\ ==0\; \end{array}$$

The function $f_{dy}$ updates the desired bytes in the buffer. If the allocated space or $f_{dy\;}\left(X\right).\text{Val}\leq Limit$ (mean of 56 bytes), then the security has been manipulated; further, if $f_{dy}\left(X\right).\text{Val}\geq Limit,$ then more space is employed from the data-link layer of the DNP3 protocol that is either real or non-existent. In the case $f_{\mathrm{dy}}\left(X\right)\cdot \text{Val}==0$, security is non-existent.

## 9. Results and Discussion

Application-layer security is essential for each type of protocol and/or communication because the bytes are constructed and the sender/response headers are deployed at the application layer. Like other SCADA protocols, the DNP3 protocol sender/response bytes are distinguished in the application layer through the employment of APCI, while the sender/responder APCI bytes are distinguished by the field designated as the IIN. The entire DNP3 protocol was designed in C#, with the use of its open library (and maximum functionalities), but due to the copyright restrictions and the future developments in the lower layers of the DNP3 protocol, the approximate performance has been measured at the application level only; as defined by the protocol, the other layers participate during the normal byte flow.

**Figure 9:**
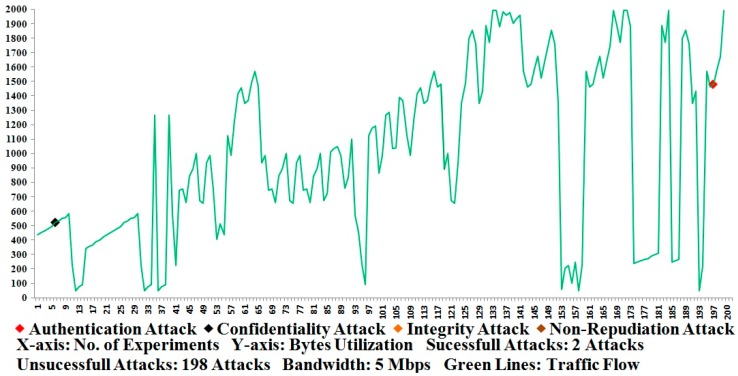
Security test using embedded DNP3 security.

**Figure 10:**
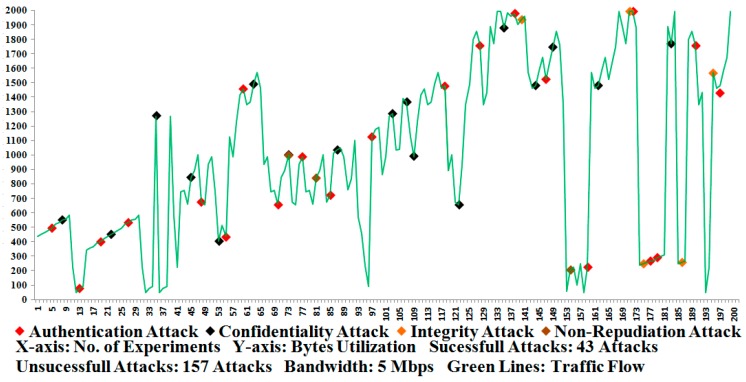
Security test using end-to-end DNP3 security.

**Figure 11:**
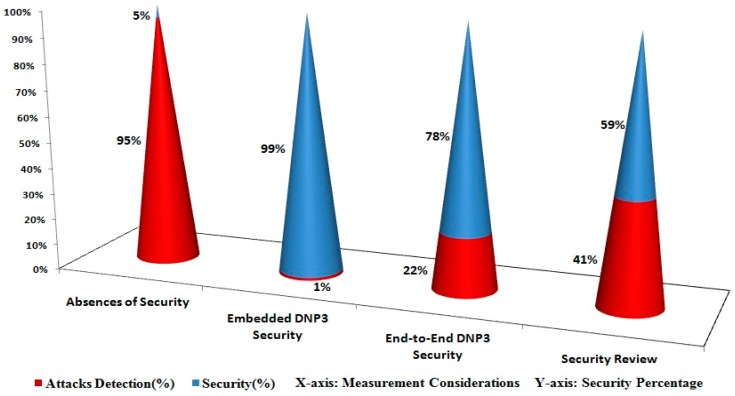
Approximate security comparison.

In the Results section, three types of considerations are taken into account regarding the measurement (*i.e.*, Figure 9, Figure 10 and Figure 11) of the most accurate performances based on our approximate knowledge. Before conducting the measurements, we ensured that the network setup (or testbed setup) and/or the participant nodes were configured properly in the testbed.

Under the first consideration, the APDU bytes were constructed and transmitted successfully several times (or 300 times) between the MTU and RTU and vice versa; here, we ensured and verified that the APDU bytes were accurately constructed and transmitted between the participant nodes. This consideration was investigated without the use of any security development, meaning that the APDU bytes were transmitted and all of the possible attacks such as Eavesdropping, Key Cracking, Man-in-the-Middle, Guessing Key (or Guessing Shared Key), Brute Force, Password Guessing, Frame Injection, Data Replay, and Data Deletion [5,56,57] were launched using built-in tools such as sniffer/dsniff, cracking tools, ethereal, ettercap, aircrack, airsnort, dinject/reinject, injection/jamming tools, and/or attack-detection mechanisms [54,55,56,57,60,61,62,63,64,65,66,67,68,69,70,71]; this resulted in an abnormal transmission (or attack transmission) for the SCADA/DNP3 system and the system performances were also measured in the absence of security development. Some attack tools are designated, however, and can be used for wireless transmissions, and we also employed and tested the testbed under the wireless-connectivity condition [72,73,74]. As far as the performances of Figure 11 show, 95 percent of attacks are successfully interrupted or detected during the transmission of APDU bytes in the absence of the proposed security development [8,61]. As a consequence, 5 percent of security has been computed from the total of 100 percent (*i.e.*, 95 percent attack detection and 5 percent security), which shows that a security mechanism that secures the SCADA/DNP3 system against potential attacks is required.

Under the second consideration, the proposed security development is used and the APDU bytes were successfully transmitted between the participant nodes on 300 occasions, and the security operations were computed at sides such as the encryption operation and the decryption operation. Each time that the APDU bytes were sent and received between the participant nodes, the encryption and decryption operations were verified by the examination that was conducted by the security development. Among the successful experiments, 200 were selected as the most favorable performance tests; further, attack scenarios were launched and 200 selected experiments were again performed with the measurements of the security [5,56,57]. Figure 9 shows the number of attacks that were observed in the testbed according to the marker indications of the byte flow that is represented by the green lines. The green lines show the flow of traffic that corresponds to the random size fragments, but each fragment is limited to 1992 bytes. As a consequence, one authentication attack and one confidentiality attack were detected and observed for a computation of the total security percentage (99 percent in the case of the embedded DNP3 security).

Under the third consideration, the security development was employed and treated as an external security development (or end-to-end security development), meaning that the proposed security development had not been a part of the DNP3 protocol, and the end-to-end system security was tested. Similar to the second consideration, the protocol bytes were successfully transmitted between the participant nodes and encryption/decryption operations were performed and verified. Subsequently, attacks such as authentication, confidentiality, integrity, and non-repudiation were launched between the transmissions, which changed the normal flow of the SCADA/DNP3 system and made the traffic abnormal [5,56,57]. Figure 10 shows the number of attacks that were observed during the end-to-end communication of the testbed. Based on the level (or number) of attacks that were detected, the security was computed as 78 percent, which is inadequate compared with the previously mentioned security percentage of 99 percent (Figure 9). Further, these results have also been compared with the existing studies [7,10,11,14,15,49,50,51,53,54] (and designated as a security review) in terms of the security level and, based on our compiled analysis, the security comparison is visualized in Figure 11.

## 10. Conclusions and Future Work

This study seeks to address the SCADA/DNP3 application-layer security challenges and issues via a cryptography mechanism, which is typically used in transmission. A secure DNP3 application-layer stack was therefore designed and the performances were measured, while formal proofs were used to evaluate the overall security implementation. The proposed security design and deployment provide new generic directions to enhance the security of the SCADA/DNP3 application layer together with future research directions. In future works, security enhancements will be made for the lower layers of the DNP3 protocol and abnormal scenarios will be developed; moreover, several attacks including authentication, integrity, non-repudiation, and confidentiality will also be tested, and the related performances will be measured to validate the proposed design and security implementation.
